# Early developmental trajectories of the impaired hand in infants with unilateral cerebral palsy

**DOI:** 10.1111/dmcn.16240

**Published:** 2025-01-18

**Authors:** Leanne Sakzewski, Susan Greaves, Ann‐Christin Eliasson, Margaret Wallen, Iona Novak, Robert S. Ware, Jill Heathcock, Nathalie Maitre, Roslyn N. Boyd

**Affiliations:** ^1^ Queensland Cerebral Palsy and Rehabilitation Research Centre, Child Health Research Centre, Faculty of Medicine The University of Queensland Brisbane Australia; ^2^ The Royal Children's Hospital Melbourne Australia; ^3^ Department of Women's and Children's Health, Neuropediatric Unit Karolinska Institutet Stockholm Sweden; ^4^ School of Allied Health Australian Catholic University North Sydney Australia; ^5^ Faculty of Medicine and Health The University of Sydney Sydney Australia; ^6^ Cerebral Palsy Alliance Research Institute, Discipline of Child and Adolescent Health, Faculty of Medicine and Health The University of Sydney Sydney Australia; ^7^ School of Medicine and Dentistry Griffith University Gold Coast Nathan Australia; ^8^ Division of Physical Therapy The Ohio State University Columbus OH USA; ^9^ Abigail Wexner Research Institute Nationwide Children's Hospital Ohio Columbus OH USA; ^10^ Emory University and Children's Healthcare of Atlanta Atlanta GA USA

## Abstract

**Aim:**

To identify developmental trajectories of impaired hand function in infants aged 3 to 15 months with unilateral cerebral palsy (CP).

**Method:**

Sixty‐three infants (37 male; median gestational age 37 weeks [interquartile range 30–39.1 weeks]) recruited as part of a randomized trial with a confirmed diagnosis of unilateral CP were included. All infants received early upper limb therapy. The Hand Assessment for Infants (HAI) was completed at baseline and until 12 to 15 months corrected age. Group‐based trajectory modelling identified groups with similar trajectories of development of impaired hand function. Multinomial logistic regression determined associations between demographic variables and trajectory membership.

**Results:**

The three‐group trajectory model comprised ‘low’ 29%, ‘moderate’ 35%, and ‘high’ 36% functioning groups. The relative risk ratio of being in the low or moderate relative to high group increased by 16% (95% confidence interval [CI] 1.02–1.32) and 14% (95% CI 1.01–1.29) respectively for each 1 week increase in gestational age. Males were more likely to be in the low relative to high group (relative risk ratio 7.22; 95% CI 1.6–32.5).

**Interpretation:**

Three distinct trajectories of development of the impaired hand were identified. Males and infants born closer to term age were at higher risk of being in a low group with little improvement over time, despite receiving early intervention.

## Graphical abstract

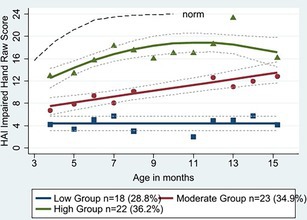

Plain language summary: https://onlinelibrary.wiley.com/doi/10.1111/dmcn.16266

**This original article is commented by Gelkop on pages** 830–831 **of this issue.**

**Abbreviations**
AHA: Assisting Hand Assessment
BIM: bimanual therapy
CIMT: constraint‐induced movement therapy
Bayley‐III: Bayley Scales of Infant and Toddler Development, Third Edition
EaHS: Each Hand Sum score
HAI: Hand Assessment for Infants
REACH: Rehabilitation very EArly for infants with Congenital Hemiplegia

**What this paper adds**

- There are three distinct trajectories of impaired hand development for infants with unilateral cerebral palsy.
- Infants born closer to term age and males are at greater risk of being in a low‐functioning group.
- A low‐functioning group demonstrated little improvement in hand function over time, despite receiving early upper limb therapy.

Infants with early asymmetric brain lesions or malformation are at risk of developing unilateral cerebral palsy (CP), where motor impairment is most apparent on the side contralateral to the brain lesion.^1^ Upper limb function is typically more affected than gross motor function. Deviation in early reaching, grasp, later manipulation, and bimanual skills are evident from 4 to 5 months post term age.^2^ Hand function and manual ability are strongly associated with later independence in activities of daily living.^3^

An international clinical practice guideline recommended the use of a suite of evidence‐based assessments to enable an accurate and early diagnosis of CP between 3 months and 12 months of age.^4^ In high‐income countries, adoption of these recommendations has seen the timing of diagnosis of high risk of CP reduce to between 4.4 to 9.5 months from 19 months of age.^5^, ^6^ Earlier diagnosis has enabled evaluation of early interventions targeting hand function in the first year of life.^2^, ^7^, ^8^, ^9^

Few studies have explored development of hand function of infants with unilateral CP. A number of studies in older children have investigated the trajectory of development of bimanual performance using the Assisting Hand Assessment (AHA)^10^, ^11^, ^12^, ^13^, ^14^ and found improvements in spontaneous use of the impaired hand between 18 months and 18 years.^10^, ^11^, ^13^, ^14^, ^15^ All studies show that children with the poorest hand function at 18 months of age demonstrate a slower rate of development, peaking between 5 to 8 years, while the highest functioning children peak earlier, at 2 years 6 months to 3 years.^12^, ^16^ These results collectively highlight the importance of early intervention to promote hand skill development for infants at high risk of unilateral CP.

We have previously described three trajectories of development of hand function based on repeated measurement on the Hand Assessment for Infants (HAI) between 3 to 12 months of age for 97 infants with unilateral CP.^9^ The largest trajectory group was a ‘low functioning’ group comprising 46% of the cohort. No associations between type of brain lesion, gestational age, or participation in motor interventions with trajectory group membership were found. One limitation of that study was that not all included infants received early targeted upper limb therapy and we were unable to determine how trajectory group membership impacted 2‐year upper limb and cognitive outcomes.^9^

This current study aimed to explore the trajectory of development of the impaired hand based on repeated measurement of the HAI in a new cohort of infants who participated in a large multi‐site randomized controlled trial. In the broader randomized controlled trial, outcomes of two infant friendly interventions commencing by 9 months of age were compared: constraint‐induced movement therapy (Baby‐CIMT) and bimanual therapy (Baby‐BIM).^7^ We found no differences on any clinical measures between the groups at the end of intervention at 12 to 15 months (depending on age at entry to the study) and 24 months corrected age; both were equally effective.^17^ This substudy will overcome one of the limitations in our previous study, in that all children in this current cohort received early targeted upper limb training. This substudy primarily aimed to determine the number and shape of trajectories of development of the impaired hand. Secondarily, we explored characteristics of trajectory group membership in this cohort of infants who all received early targeted upper limb training. Understanding the factors that are associated with different trajectories of development of hand function of infants at high risk of unilateral CP may lead to the development of different interventions targeted to those factors/characteristics. Finally, we explored differences in the upper limb and cognitive outcomes for each trajectory group at completion of the intervention (12–15 months corrected age) and at 24 months corrected age. Compared to our previous study, we expected there would be fewer infants in the low trajectory group as all infants received early intervention.

## METHOD

Ethical approvals were obtained from sites in the Rehabilitation very EArly for infants with Congenital Hemiplegia (REACH) clinical trial in Australia (Brisbane, Perth, Melbourne, and Sydney) and the USA (Ohio and Minnesota). The trial was registered with the Australian New Zealand Clinical Trials Registry (ACTRN12615000180516). Details of the study are reported in the trial protocol.^7^

### Participants

Participants in this longitudinal cohort study were recruited as part of the larger REACH trial.^7^ In the larger REACH study, 96 infants identified at high risk of unilateral CP were recruited between 2016 and 2020. To be eligible for inclusion in the REACH trial, infants were (1) aged between 3 to 9 months corrected age at time of study entry, (2) with asymmetric brain lesions (identified on cranial ultrasound or magnetic resonance imaging). They had (3) absent fidgety movements on the General Movements Assessment at 12 to 16 weeks corrected age or abnormal Hammersmith Infant Neurological Examination (between 18–39 weeks corrected age), and (4) asymmetric upper limb object‐related hand use on the HAI of more than 3 points between limbs. To be included in this present substudy, infants had a confirmed diagnosis of unilateral CP by a medical practitioner (paediatrician, rehabilitation physician, neonatologist, or neurologist) between 12 to 24 months corrected age and two or more assessments on the HAI. Infants with a diagnosis of bilateral CP or no CP and those with a single assessment timepoint on the HAI were excluded.

### Study interventions

After screening for eligibility and baseline assessments, infants were randomized using concealed allocation to either receive Baby‐CIMT or Baby‐BIM. Full details of the interventions are in the study protocol.^7^ In brief, both interventions were implemented by parents in the home environment. An occupational therapist or physiotherapist, who completed standardized training in both approaches, conducted monthly home visits from the time of recruitment until 12 to 15 months corrected age depending on the age of the infant when they entered the study (total 6–9 home visits). Two weeks after each home visit, the therapist contacted the family by telephone, Zoom, or Skype to provide additional support. Parents were coached by the therapist to deliver the intervention 5 days per week, aiming for 20 minutes per day between 3 to 6 months corrected age, 30 minutes between 6 to 9 months corrected age, and 40 minutes for infants 9 months and older.

Baby‐CIMT involved constraint of the unimpaired hand using a glove, sock, or sleeve with a bag clip, accompanied by unimanual play targeted at the child's ability level. Baby‐BIM comprised play‐based activities designed to provoke use of both hands during bimanual activities through careful selection of toys/activities and based on the child's ability level.

### Data collection

Data were collected as part of screening, baseline, and follow‐up assessments during home visits conducted by study therapists. Assessments were videorecorded and later scored by certified occupational therapists who were blinded to group allocation and timing of assessments.

### Outcome measures

#### Hand Assessment for Infants

The HAI is a criterion and norm referenced measure of object‐related hand use for infants at risk of developing unilateral CP, aged from 3 to 12 months post term.^18^, ^19^ This observation‐based measure uses a semi‐structured play session with age‐appropriate toys presented in specific locations and positions to elicit unimanual and bimanual hand use. The assessment is videorecorded for later standardized scoring by a certified rater. The HAI comprises 12 unimanual items scored separately on each hand and five bimanual items, all rated on a 3‐point scale. In this study we used the Each Hand Sum scores (EaHS). EaHS for the impaired upper limb are reported as the sum of raw scores and range from 0 to 24.^18^ The HAI has excellent interrater and test–retest reliability for EaHS (intraclass correlation coefficients ranging between 0.96 and 0.99).^20^ The HAI was performed as part of the screening process to determine eligibility at study entry between 3–9 months corrected age, then at 6 months corrected age for infants who commenced the intervention before 6 months corrected age and at the conclusion of the intervention (12–15 months corrected age). The HAI was used for a number (*n* = 25) of infants older than 12 months to enable a single measure of pre‐post intervention for those entering the study after 6 months corrected age. We chose the EaHS for the impaired hand rather than overall HAI units as a previous early Baby‐CIMT study demonstrated changes on the EaHS,^2^ and we expected that all infants would achieve age‐appropriate EaHS for the unimpaired hand.

#### Mini‐Assisting Hand Assessment and Assisting Hand Assessment

The Mini‐AHA is a criterion‐referenced, Rasch developed measure that evaluates how children with unilateral CP, aged 8 to 18 months, use their impaired hand in activities that provoke bimanual performance.^21^ It is a semi‐structured play session and comprises 20 items rated on a 4‐point scale. Construct and internal scale validity have been established by test developers.^21^ The Mini‐AHA was completed at the end of the intervention period (12–15 months corrected age). The AHA 5.0 was completed at 24 months corrected age.^22^ The AHA 5.0 evaluates how effectively a child uses their impaired hand in bimanual activities. Scores are reported as logit‐based AHA units. The 20‐item version has excellent internal scale validity.^22^ The AHA and Mini‐AHA are reported on a 0 to 100 scale with higher scores indicating better bimanual performance.

#### Bayley Scales of Infant and Toddler Development, Third Edition

The Bayley Scales of Infant and Toddler Development, Third Edition (Bayley‐III) is a norm‐referenced measure of cognitive, motor, language, and social–emotional development for children aged 0 to 3 years 6 months. Infants were assessed between 12 to 15 months at the conclusion of the intervention and at 24 months corrected age. Domain quotient scores are reported with increasing numbers reflecting better performance.^23^ The cognitive domain was used in this study.

### Statistics

Cohort characteristics were summarized using mean and standard deviation (SD) or median and interquartile range (IQR) for continuous data depending on data distribution and frequency and percentage for categorial variables. Group‐based trajectory modelling was performed using the ‘traj’ plugin in Stata^24^ and a censored normal model to describe subgroups of infants who displayed similar trajectories of performance of the impaired hand, measured on the HAI EaHS. Models were systematically fitted, ranging from one to four groups and first order (linear) to third order (cubic) models. As per recommendations, models were excluded if one or more trajectory group comprised less than 5% of the cohort.

Models were compared using Bayesian information criteria. Posterior probability was calculated to determine the likelihood of an infant belonging to a group, and the final model was evaluated using average posterior probabilities of group membership, odds of correct classification, and proportion of infants assigned to each trajectory group. Multinomial regression was used to examine univariable associations between demographic variables and trajectory group membership. After finalization of trajectory modelling, bimanual performance measured on the Mini‐AHA at 12 to 15 months and AHA at 24 months, and cognition (Bayley‐III) were summarized for trajectory groups and compared using Tukey's range test. Statistical analyses were performed using Stata 17.0 (StataCorp LP, College Station, TX, USA).

## RESULTS

Of the 96 infants recruited to the larger randomized control trial, 63 with a confirmed diagnosis of unilateral CP at 12 to 24 months of age were included in this substudy. The median gestational age at birth was 37 weeks (IQR 30–39.1 weeks), 37 (57%) were male, 34 (54%) received Baby‐CIMT, and 29 (46%) received Baby‐BIM. Infants were assessed on the HAI either two (*n* = 31) or three times (*n* = 32) depending on whether they commenced before or after 6 months corrected age. Overall, there were 157 observations with mean (SD) of 2.5 (0.6) per participant.

### Developmental trajectories of impaired hand function

**FIGURE 1:**
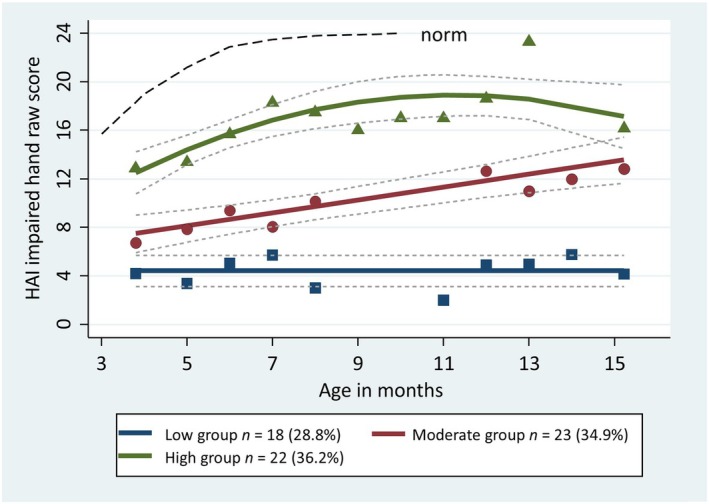
Group‐based trajectory model of development of the impaired hand (Hand Assessment for Infants [HAI]) in infants with unilateral cerebral palsy between 3 months and 15 months of age. Coloured dots and dotted line represent means and 95% confidence intervals. The black dotted line represents normative values (from Ek et al.).^19^

Demographic characteristics of the entire sample and each trajectory group are summarized in Table 1. Figure 1 depicts the fitted trajectories for a three‐group model and coefficients are summarized in Table 2. There was an appropriate proportion of infants (>5%) in each group: 18 (29%) in the low‐functioning group, 22 (35%) in the moderate‐functioning group, and 23 (36%) in the high‐functioning group. A good model fit (Table 3) was indicated with the mean posterior probabilities ranging from 0.89 to 0.98 (exceeding recommended 0.70), odds of correct classification between 14.5 and 74.3 (exceeding recommended 5).^25^

**TABLE 1:** Demographic characteristics of total sample of included infants and by trajectory group.

	Total group (*n* = 63)	Group 1 ‘Low group’ (*n* = 18)	Group 2 ‘Moderate group’ (*n* = 23)	Group 3 ‘High group’ (*n* = 22)
Sex, male *n* (%)	37 (59)	15 (83)	13 (57)	9 (41)
Right side unilateral CP, right *n* (%)	36 (57)	12 (67)	14 (61)	10 (45)
Gestational age at birth (weeks), (median, IQR)	37 (30, 39.1)	37 (35.5, 39.1)	38 (29.2, 40)	30.4 (29.2, 37.2)
Preterm birth				
< 28 weeks	6 (9)	1 (5)	2 (9)	3 (14)
28–31 weeks	17 (27)	3 (17)	4 (17)	10 (45)
32–36 weeks	8 (13)	3 (17)	2 (9)	3 (14)
≥ 37 weeks	32 (51)	11 (61)	15 (65)	6 (27)
Brain lesion laterality				
Unilateral *n* (%)	42 (72)	14 (78)	17 (74)	11 (65)
Bilateral *n* (%)	16 (28)	4 (22)	6 (26)	6 (35)
Treatment				
Baby‐CIMT, *n* (%)	34 (54)	6 (33)	14 (61)	14 (64)
Baby‐BIM, *n* (%)	29 (46)	12 (67)	9 (39)	8 (36)
Dose (hours), mean (SD)	51.1 (19.6)	54.9 (16.9)	53.1 (20.3)	46.2 (30.5)
Baseline, mean (SD)				
HAI EaHS impaired	9.1 (5.3)	4.2 (2.4)	7.5 (3.1)	14.7 (3.4)
HAI EaHS unimpaired	21.2 (2.9)	19.4 (4)	21.4 (2.3)	22.1 (2)
HAI asymmetry	57.9 (22.9)	77.6 (15.0)	65.8 (12.6)	33.6 (13.3)
HAI units	51.7 (11.0)	42.3 (7.4)	49.3 (6.6)	61.9 (8.9)
Final, mean (SD)				
HAI EaHS impaired	12.2 (6.3)	4.2 (1.8)	12.6 (1.9)	18.7 (3.3)
HAI EaHS unimpaired	23.8 (0.8)	23.9 (0.5)	23.7 (1.2)	23.9 (0.4)
HAI asymmetry	48.6 (26.5)	82.5 (8.0)	46.7 (7.7)	21.6 (14.5)
HAI units	63.3 (13.9)	48 (3.0)	62.1 (4.8)	77.8 (10.9)
Mini‐AHA 12–15 months, mean (SD)	*n* = 62 52.3 (22.3)	*n* = 18 25.9 (9)	*n* = 23 51.7 (8.8)	*n* = 21 75.6 (12.7)
AHA 24 months, mean (SD)	*n* = 57 54.8 (21)	*n* = 14 28.1 (13)	*n* = 23 53.5 (10.2)	*n* = 20 75.1 (10.1)
Bayley‐III cognitive 12–15 months, median (IQR)	*n* = 58 93.8 (14.8)	*n* = 15 80 (75, 95)	*n* = 23 95 (85, 105)	*n* = 20 97.5 (95, 100)
Bayley‐III cognitive 24 months, median (IQR)	*n* = 56 90 (80, 95)	*n* = 13 85 (80, 95)	*n* = 22 90 (80, 95)	*n* = 21 90 (85, 95)

Abbreviations: AHA, Assisting Hand Assessment; BIM, bimanual intensive therapy; Bayley‐III; Bayley Scales of Infant and Toddler Development, Third Edition; CIMT, constraint‐induced movement therapy; CP, cerebral palsy; EaHS, Each Hand Sum score; HAI, Hand Assessment for Infants; IQR, interquartile range.

**TABLE 2:** Maximum likelihood estimates for group‐based trajectory model of impaired hand function in infants with unilateral CP.

	Term	HAI EaHS impaired hand (*n* = 157) BIC: −468.53		
Trajectory group		MLE	SE	*p*
1	Intercept	4.32	0.49	< 0.001
2	Intercept	5.45	1.08	< 0.001
	Linear	0.53	0.11	< 0.001
3	Intercept	4.27	2.84	0.14
	Linear	2.60	0.70	< 0.001
	Quadratic	−0.12	0.04	0.003
		% group membership		
1		28.8		
2		34.9		
3		36.2		

Model selection: three group model with two linear and one quadratic trajectories (1, 2).

Abbreviations: BIC, Bayesian Information Criteria; CP, cerebral palsy; EaHS, Each Hand Sum score; HAI, Hand Assessment for Infants; MLE, maximum likelihood estimation; SE, standard error.

**TABLE 3:** Average posterior probability value, odds of correct classification, estimated group probabilities, and the proportion of the sample assigned to the group.

	HAI EaHS impaired hand trajectory group (*n* = 63)		
	Low‐functioning	Moderate‐functioning	High‐functioning
Average posterior probability	0.95	0.89	0.98
Odds of correct classification	46.2	14.5	74.3
Estimated group probability	0.29	0.37	0.35
Proportion assigned to group according to maximum posterior probability assignment rule	0.29	0.35	0.36

Average posterior probabilities greater than 0.70 and odds of correct classification greater than 5 are recommended and indicate good model fit.

Abbreviations: EaHS, Each Hand Sum score; HAI, Hand Assessment for Infants.

The largest proportion of infants were in the high‐functioning group, followed by a moderate‐functioning group. The high‐functioning group appeared to decline slightly at the final assessment point. Four infants were 1 point lower at the final assessment, one infant was 2 points lower, and one was 3 points lower. The most common HAI items that had reduced scores were ‘initiates use for objects on the side’, ‘initiates use for objects in the midline’, and ‘moves forearm’ or ‘moves upper arm’. Infants in the moderate‐functioning group had a consistent and upward trajectory. Infants in the low‐functioning group demonstrated little change in HAI EaHS for the impaired hand over time.

Associations were found between trajectory membership and gestational age and sex (Table 4). With every 1‐week increase in gestational age, the relative risk of being in the low‐functioning group increased by 16% and in the moderate‐functioning group by 14% relative to the high‐functioning group. Males were at a seven times greater risk of being in the low‐functioning group relative to the high‐functioning group. There were no associations between trajectory group membership and type of intervention, dose, side of unilateral CP, or brain lesion.

**TABLE 4:** Relative risk ratios and 95% confidence intervals from univariate multinomial logistic regression showing correlates of low‐functioning and moderate‐functioning groups versus the high‐functional group (reference group).

	Low‐functioning group			Moderate‐functioning group		
	RRR	95% CI	*p*	RRR	95% CI	*p*
Gestational age	**1.16**	**1.02–1.32**	**0.03**	**1.14**	**1.01–1.29**	**0.03**
Sex (male)	**7.22**	**1.60–32.50**	**0.01**	1.88	0.57–6.14	0.30
Brain lesion (bilateral)	0.52	0.12–2.33	0.40	0.65	0.17–2.53	0.53
Side of unilateral CP (right)	2.4	0.66–8.72	0.18	1.87	0.57–6.11	0.30
Dose	1.02	0.99–1.06	0.18	1.02	0.99–1.05	0.24
Treatment (Baby‐BIM)	0.33	0.09–1.19	0.09	0.83	0.24–2.78	0.76

Bold type denotes a significant association.

Abbreviations: BIM, bimanual intensive therapy; CI, confidence interval; RRR, relative risk ratios.

The high‐ and moderate‐functioning group had significantly higher scores on the Mini‐AHA at 12 to 15 months corrected age compared to the low‐functioning group (mean difference 49.7, 95% CI 43.0–56.3; *p* < 0.001 and mean difference 25.7, 95% CI 19.2–32.2; *p* < 0.001 respectively). At 24 months corrected age, the low‐functioning group had significantly poorer scores on the AHA compared to the moderate‐functioning (mean difference 25.4, 95% CI 17.9–32.8; *p* < 0.001) and high‐functioning groups (mean difference 46.9, 95% CI 39.3–54.5; *p* < 0.001). Infants in the low‐functioning group had poorer scores on the Bayley‐III cognitive domain compared to the high‐functioning group at 12 to 15 months corrected age (mean difference 13.1, 95% CI 3.5–22.6; *p* = 0.008). By 24 months corrected age, there were no significant differences between trajectory groups on the Bayley‐III cognitive domain.

## DISCUSSION

This study of the impaired hand in a cohort of infants with unilateral CP who all received early, targeted upper limb intervention identified three distinct trajectories of longitudinal development. The high‐ and moderate‐functioning groups demonstrated increased function over the study period. The low‐functioning group demonstrated limited change in their impaired hand function over time coupled with lower developmental test scores, despite receiving targeted early intervention. Males and infants born closer to term equivalent age had a greater risk of being in the low‐functioning relative to the high‐functioning group. The results indicating males are at a greater risk should be viewed with caution because of the wide 95% CIs.

Our previous study modelled development of hand function using the total score (HAI units) rather that the EaHS, so it is not possible to directly compare results and groups. While the EaHS and HAI units are different, poor EaHS scores will be reflected in lower HAI unit scores. We were particularly interested in the EaHS trajectory given that all infants received targeted upper limb therapy using either a bimanual or unimanual approach. This differs from our previous study in which only 41% of infants received therapy.^9^ In the earlier study, the proportion of infants in the low‐functioning group was larger, comprising 46% of the cohort. We expected that we would have a smaller proportion of children in a low‐functioning group, given all received early intervention and at a higher dose than those in the previous study. There may be a number of factors contributing to this difference. First, 49% of the cohort in this current study were born preterm which is a greater proportion than population‐based estimates^26^, ^27^ compared to our previous cohort which was over‐represented by infants with perinatal stroke.^9^ Studies investigating the relationship between timing of brain injury and upper limb function have demonstrated that children with earlier onset injuries (e.g. periventricular lesions, predominantly white matter) have better motor and sensory function than those with later onset injury (e.g. cortical/subcortical lesions, predominantly grey matter).^28^, ^29^, ^30^ Our findings support this, as infants in the high‐functioning group were born significantly earlier than those in the low‐functioning group. Second, in our previous study, fewer infants received targeted upper limb intervention and at a lesser dose compared to our current study which may account for the larger proportion of infants in the low‐functioning group.

The finding that males are at greater risk of being in the low‐functioning relative to the high‐functioning group is somewhat inconsistent with the literature and should be treated cautiously because of the estimate imprecision. It is acknowledged that CP more frequently occurs and the prevalence rate is up to 30% higher in males compared to females.^31^ This biological vulnerability in males has been explained by factors such as genetic predisposition,^32^ differences in brain organization,^33^ and the presence, in females, of female hormones mediating the consequences of brain damage.^32^ In a recent narrative review of sex differences in CP, findings from 70 included papers confirmed that males had a greater biological vulnerability in developing both mild and severe CP although there was inconsistent evidence that sex impacted severity of impairment, performance, or outcomes of intervention.^31^ The authors noted, however, that despite most studies including both males and females, very few explored or reported sex differences. Also, few studies included infants under 2 years of age, limiting generalization of findings to the current study.^31^

The clinical profile of infants in each trajectory group mirrors our previous findings.^9^ The ability to grasp by 6 months of age is a clinical feature distinguishing the three groups, with the low‐functioning group demonstrating early small movements of the impaired upper limb developing to the inconsistent ability to contact, but not grasp, toys/objects. This suggests that very early intervention should focus on training the acquisition of grasp, since grasp by 6 months is strongly predictive of long‐term outcomes. In addition, the acquisition of grasp by 6 months could be a potentially important biomarker and outcome indicator for very early intervention studies. By 5 months of age, the profile of the moderate‐functioning group was distinctly different to the low‐functioning group, with the moderate group starting to develop active grasping of toys/objects, at a similar age as the previous study.^9^ It is possible that the low‐functioning group requires a different approach to early intervention. This might include different ways to help infants learn strategies to enhance successful experiences with their affected hand. Although the high‐functioning group had the ability to grasp by 6 months of age, upper limb function in this high‐functioning group remained greater than two standard deviations below the normative sample throughout infancy.^19^ We have now extended on these findings to evaluate later bimanual performance and cognitive functioning at 24 months corrected age. Our findings still demonstrate that there are still significant differences between trajectory groups at 12 to 15 months and 24 months corrected age.^16^ The significantly poorer performance on the Bayley‐III cognitive domain at 12 to 15 months was not evident at 24 months. This requires further exploration, as it is unclear if and how cognition impacts an infant's ability to use two hands together in early play.

This study uniquely identified the association between gestational age and sex on the trajectory of development on the impaired hand in infants with unilateral CP. The study, however, has a number of limitations. The HAI is only validated for infants with unilateral CP up until 12 months of age,^18^ and a small number of our cohort were aged up to 15 months corrected age at time of assessment. Although these infants did not reach the ceiling of the HAI, suggesting that the result are valid, it is unclear whether age at assessment may have influenced the modelling of hand trajectories. The decline in the highest functioning group is not simple to understand and has to be further explored. The specific items that appeared to decline in scores over time may suggest that as the infants got older, the speed and ease of use of the preferred hand may have influenced whether and how quickly they initiated use of their affected hand. Additionally, increasing spasticity may have influenced range and ease of upper limb movements. Another limitation is the relatively small number of assessments at each age in trajectories. Given variable ages at study entry, it was impossible to standardize the timing of measurements. One challenge is the absence of a single measure of hand function from infancy through to childhood. It may be possible to link the Mini‐AHA and AHA scores through Rasch measurement modelling, as the items and scale are consistent across the measures.^21^, ^34^ The possibility of linking the HAI to the Mini‐AHA is probably not possible, given the different scales (3 points on HAI, 4 points on Mini‐AHA) and different concept of the assessments, HAI investigates both hands separately and together, and Mini‐AHA explores the ability to use the impaired hand in bimanual activities.^18^, ^21^

### Conclusion

Three trajectories of development of the impaired upper limb were identified in a cohort of infants with unilateral CP who all received targeted upper limb intervention in infancy. Children in the high‐functioning group had higher scores at 4 months than both the moderate‐ and low‐functioning groups but had similar scores (overlapping CIs) to the moderate‐functioning group at 15 months. The low‐functioning trajectory group showed little change over time.^9^ This group poses a challenge for researchers and clinicians to identify, tailor, and implement interventions to help infants learn strategies to optimize functional hand use and future performance of activities in daily life. Future studies would benefit from standardized timing of assessments in order to provide more nuanced information on trajectories based on age and gestational age, which may assist in predicting outcomes and future resource utilization.

## Data Availability

The data that support the findings of this study are available from the corresponding author upon reasonable request.
